# What medical specialists want to stay in remote areas of Indonesia: Discrete choice experiments

**DOI:** 10.1371/journal.pone.0308225

**Published:** 2024-08-15

**Authors:** Anna Kurniati, Ferry Efendi, Angger Rina Widowati, Agustina Simanjuntak, Siska Mudina, Budi Ikhwansyah, Arif Yustian Maulana Noor, Lisa McKenna

**Affiliations:** 1 Directorate General of Health Workforce, Ministry of Health, Jakarta, Indonesia; 2 School of Nursing and Midwifery, La Trobe University, Melbourne, Australia; 3 Faculty of Nursing, Universitas Airlangga, Surabaya, Indonesia; 4 Research Excellent in Advancing Community Healthcare (REACH), Universitas Airlangga, Surabaya, Indonesia; 5 Agriculture Socio-Economic Department, Faculty of Agriculture, Universitas Brawijaya, Malang, Indonesia; Universitas Pelita Harapan, INDONESIA

## Abstract

**Introduction:**

The equitable distribution of medical specialists in Indonesia’s remote areas remains a challenge. This study investigated the preferences of medical specialists regarding retention programs aimed at addressing this issue.

**Methods:**

A Discrete Choice Experiment (DCE) was utilized to collect stated preferences from 341 medical specialist working in district general hospitals across 10 Indonesian provinces. The DCE retention questionnaire focused on eight key characteristics: location, medical facilities, net income, continuing professional development program, security, length of commitment, source of incentives, and caseload.

**Results:**

The study found that the most influential factors for retention in remote areas were security guarantees from the local government (OR = 6.11), fully funded continuing professional development programs (OR = 2.84), and access to advanced medical facilities (OR = 2.35).

**Conclusion:**

The findings indicate that a comprehensive intervention package, with a particular emphasis on security provisions, is necessary to retain medical specialists in remote areas. Financial incentives are also recommended to improve retention. However, it is crucial to acknowledge that no single intervention will suffice, as the factors influencing specialist retention in remote areas of Indonesia are complex and multifaceted.

## Introduction

Medical doctors and specialists play an essential role among the larger group of health care workers (HCWs) [1]. However, an uneven distribution of medical specialist can negatively impact the efficiency and sustainability of healthcare systems [2]. Lack of medical specialists or medical doctors is a persistent issue in most remote areas, both regionally and globally, with substantial repercussions for the quality, scope, and equity of access to services [3]. Additionally, it limits local residents’ access to health services [4, 5]. The strategic allocation of medical specialists is crucial for enhancing the effectiveness of the healthcare system and accomplishing important health goals, especially in low- and middle-income countries [6–8]. Many countries have implemented strategies to retain medical specialists in remote areas, but results have varied [9]. While there is international evidence demonstrating the effectiveness of these strategies in diverse economic contexts, from low-income to high-income countries [7, 8, 10], this research focuses on the adaptation and implementation of these strategies in Indonesia’s unique context. Recognizing and understanding local factors is critical to designing successful retention strategies in the Indonesian healthcare landscape.

Despite the World Health Organization (WHO) setting a target doctor-to-population ratio of 1:1,000, more than 44% of WHO Member States reported doctor-to-population ratios that were lower than this benchmark. In other words, there was less than one doctor for every 1,000 people in more than 44% of Member States [11]. Based on the planning of health workers by Indonesia’s Ministry of Health, the target ratio of basic medical specialists includes 0.024 pediatricians per 1,000 population, internal medicine specialists 0.03 per 1,000 population, 0.02 obstetrics and gynecology specialists per 1,000 population, and 0.02 surgeons per 1,000 population. Meanwhile, the target ratio of medical specialists according to the health workforce utilization plan in 2025 is 0.12 per 1,000 people [12]. The ratio shows that the need for medical specialists has not reached the expected target ratio. If we compare the number of registered medical specialists with population and areas by region in Indonesia, it can be seen that the islands of Sumatra, Java-Bali and Sulawesi still have the highest ratios of medical specialists. The ratio of medical specialists to 1,000 people based on region are respectively Sumatra at 0.13, Java-Bali at 0.17, Kalimantan at 0.10, Sulawesi at 0.15, and Maluku-East Nusa Tenggara-Papua at 0.05 [12]. Data shows that the highest ratio is still in the Java-Bali region. This contrast with the situation in eastern Indonesia, which still faces a shortage of medical specialists due to uneven distribution. The overall number of medical specialists in Indonesia in 2021 was 40,320 people. Meanwhile, the need for specialist physicians according to the 2021–2024 national defense system reform is 75,657 people. Based on these data, it has been identified that the shortage of medical specialists was 35,337 people for 2021–2024 [12].

Retention of medical specialists in remote areas remains a challenge in Indonesia. The Indonesian Ministry of Health is committed to accelerating their distribution as mandated in the country’s health transformation [12]. The acceleration of health worker deployment aims to boost workforce capacity, particularly in remote areas where healthcare services remain underserved [12–14]. The crisis for health workers in remote areas risks is hampering national development, especially the target of realizing universal health coverage for all Indonesians [15]. The Indonesian Government has employed various strategies to deploy and retain medical specialists in remote areas, including civil service recruitment, non-permanent employee recruitment, and specialized assignments such as ’Penugasan Khusus’ (special assignment) programs [16]. Another mechanism involves contractual support through the Medical Specialist Education Program for in-country medical specialist education. However, these efforts are temporary and ineffective for long-term medical specialist retention [12].

Another program recently launched by the Indonesian Ministry of Health, the Academic Health System (AHS), aims to integrate the education and health systems [17]. This integration is intended to achieve an even distribution of medical specialists. The government identifies medical specialist needs in specific areas in accordance with certain regulations addressing Hospital Classification, considering factors critical for retention, such as willingness to serve in specific conditions. Mechanisms for appropriately assigning medical specialists include AHS’s integration of Teaching Hospitals, medical faculties, and other institutions, enhancing public health service quality through coordinated education, research, and service delivery, thus addressing regional specialist shortages and improving healthcare access [18, 19].

The issue of medical specialist retention in remote areas needs to be a major focus, particularly for government efforts to achieve universal health coverage. The challenge of retaining medical specialists in remote areas must be a primary focus to attain universal health coverage [20]. Various factors have been reported to impact the decisions of medical specialists to work and thrive in remote areas, encompassing location [21], security [22, 23], medical facilities, income [24], transportation facilities, housing facilities [25], educational support [26], and incentives [14].

Discrete Choice Experiment (DCE) approach has been widely applied in the health sector, particularly to understand the preferences of health workers when making choices between two or more alternatives [27, 28]. Earlier research in Ethiopia [29] utilized DCE to gauge the factors influencing health worker decisions. In Western Kenya [30], a study identified the motivations of community health workers for retention, highlighting both material and non-material incentives. Furthermore, research in Malang district, Indonesia [31], explored the employment preferences of healthcare workers, offering valuable insights for policy interventions. A recent DCE study from Indonesia examines the retention of doctors and dentists in remote areas of Indonesia, exploring factors influencing their decision to stay and strategies to improve retention rates [32]. More specifically, a 2020 study in Turkey focused on job attribute preferences of physicians and nurses, including specialists, employing DCE. The results concluded that these DCE findings could guide policymakers in understanding the characteristics that enhance the motivation of healthcare professionals to work in rural areas in Turkey [33].

Previous research has not discussed the preferences of medical specialists working in remote areas, especially in the Indonesian context. In this study, we aimed to specifically focus on medical specialist preferences for retention in remote areas. By conducting DCE, we seek to address the gaps and limitations identified in previous research and provide a deeper understanding of the factors most favorable to medical specialist retention in remote areas. It is hoped that the results of our research will provide insights into policy options that can be implemented by the government to increase the retention of medical specialists in underserved areas. The results of the current study were expected to describe preferences of medical specialists to be retained in remote areas and provide information for the government regarding policy options that can be implemented. Hence, this study aimed to analyze retention of medical specialists in remote areas using the DCE approach.

## Materials and methods

### Study design and setting

The research utilized the DCE approach which is a quantitative method for analyzing preferences and job choices of medical specialists [7, 28]. The study was conducted from November 11th to November 24th, 2022, in 10 provinces, with five regencies in each province, as defined by Presidential Decree No. 63 of 2020 regarding the designation of remote areas. Starting from the provinces of East Nusa Tenggara, Maluku, North Sumatra, Papua, West Papua, Central Sulawesi, West Sumatra, North Maluku, Lampung and West Nusa Tenggara. In Indonesia, a regency is an administrative region or administrative subdivision, similar to a county or district in other countries. We collected data at 48 district general hospitals in these provinces for approximately one month. These hospitals were selected to ensure diversity in terms of location and demographics of respondents, allowing for a comprehensive representation of the healthcare landscape in these regions.

### Attribute development

The initial step of identifying attributes and levels was carried out through literature review and focus group discussion (FGD). A literature search for studies was conducted through a variety of academic databases namely Scopus, Web of Science, Pubmed, EBSCO, and Proquest using keywords retention AND remote areas AND Discrete Choice Experiment OR DCE AND medical specialist OR specialist physician OR specialist. The DCE attribute was developed by taking into account the indicators of medical specialist retention. The FGD included invited stakeholders such as the Director for Health Worker Empowerment, the National Research and Innovation Agency, the Health Development Policy Agency, the main DCE research team, and invitees. The invitees were representatives of medical specialists who were currently or had previously worked in remote areas for at least one year, comprising a total of five specialists. The FGD involved participants learning about remote health services and ongoing strategies for retaining medical specialists. Following that, participants shared their thoughts on these strategies, drawing on their personal experiences and knowledge. Participants then deliberated on each attribute through collaborative discussions until a consensus was reached for the final version of the DCE attributes. The FGD produced eight attributes, each with 2–3 levels, which were then converted into an instrument study questionnaire. Table 1 provides a more detailed breakdown of research levels and attributes.

**Table 1:** Retention attributes, levels, and operational definitions.

Attribute	Level	Operational definition
Location	Not a remote area ^R^	Regency areas whose territories and people are developing compared to other regions on a national scale.
	Remote area	Regency areas whose territories and people are less developed compared to other regions on a national scale.
Medical facilities (medicines and medical devices)	Inadequate ^R^	Medical facilities such as medicines and medical devices are inadequate in supporting the delivery of promotive, preventive, curative and rehabilitative health services.
	Adequate to standard	Availability of health service facilities as well as pharmaceutical products and medical devices according to standards in supporting the implementation of promotive, preventive, curative and rehabilitative health services.
	More than standard	Availability of health service facilities as well as more than standard pharmaceutical products and medical devices, such as networks and systems to support the delivery of promotive, preventive, curative and rehabilitative health services.
Source of incentives	Regional Incentives/ Central Incentives ^R^	Government assistance provided to increase income outside of salaries/wages and the welfare of medical specialists in carrying out their duties.
	Medical services or services	Services are rewards for services provided for administrative actions and or other services. Medical services are compensation for services provided by medical specialist directly to patients in the context of carrying out observations, diagnoses, treatment, consultations, visits, actions/manovers/maneuvers, medical rehabilitation, and/or other services.
	All incentives	Additional regional incentives/central incentives and incentives for medical services/services provided to increase work enthusiasm.
Continuing Professional Development Program	Unfunded ^R^	No financial assistance is provided in participating in the sustainable development program.
	Fully funded	Provided full cost assistance in participating in sustainable development programs.
	Partially funded	Provided assistance with partial costs in participating in sustainable development programs.
Security	No security guarantee from the government ^R^	There is no guarantee for the security and safety of medical personnel from the government in providing health services, especially in remote and very remote areas.
	Security guarantee from the local government	There is a guarantee of security and safety from the local government in carrying out health services, especially in remote and very remote areas.
	Guaranteed security from indigenous peoples/local communities	There is a guarantee of security and safety from indigenous peoples/local communities.
Length of commitment	2 years ^R^	Committed to work in the same place for 2 years and not allowed to move to another place during the assignment period.
	4 years	Committed to work in the same place for 4 years and not allowed to move to another place during the assignment period.
	Until retirement	Committed to work in the same place until retirement and are not allowed to move to another place.
Number of cases handled	A little ^R^	The number of patients served is not more than 1000 cases/year.
	Enough	The number of patients served is 1000 to 3000 cases/year.
	Lots	The number of patients served is more than 3000 patients per year.
Net income (IDR)	35 million	The income earned includes basic salary, position allowance/service allowance, welfare allowance, and other benefits with a total of 35 million every month.
	75 million	The income earned includes basic salary, position allowance/service allowance, welfare allowance, and other benefits with a monthly total of 75 million.
	100 million	Income earned includes basic salary, position allowance/service allowance, welfare allowance, and other benefits with a monthly total of 100 million.

Note: ^R^ = Reference level; The IDR to USD exchange rate is IDR 15,506 as of December 7, 2022

### DCE design and piloting

**Fig 1:**
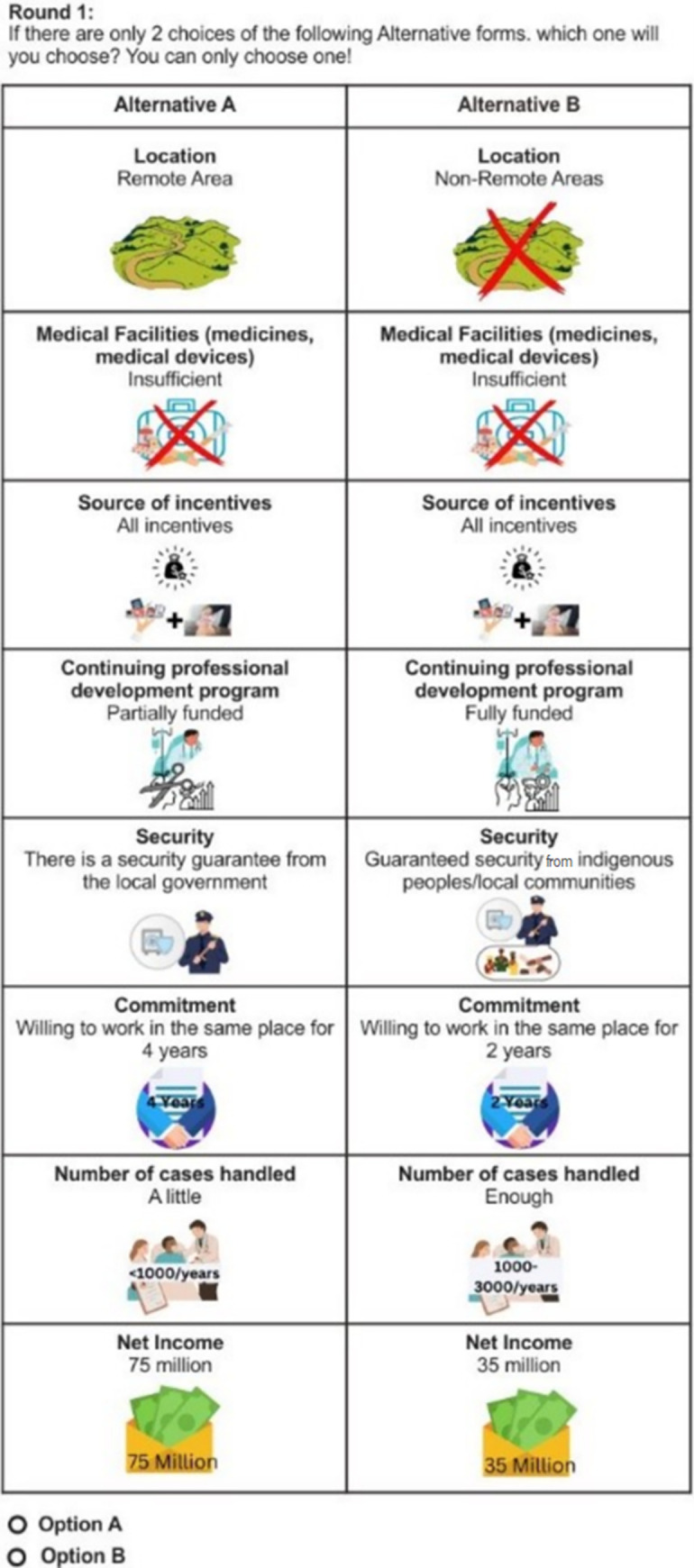
One of the choice sets in the study.

The paper-based questionnaire for the DCE retention study was designed using package support.CEs in the R software [34] (Fig 1). Due to the large number of possible combinations, an orthogonal fractional factorial design was used to generate 18 choice sets with two alternatives each [35]. We developed the questionnaire with rigor in mind to overcome the content validity issue based on WHO guideline [36]. A panel of subject matter specialists, including the Director for Health Worker Empowerment, the National Research and Innovation Agency, the Health Development Policy Agency, and medical specialists, carefully examined the original concept. In addition, the 30-health worker pilot test’s objective was to further validate the material by getting feedback on the questionnaire’s attributes and levels’ relevancy and clarity. These steps ensured the validity of the instrument content.

### Population and sampling

The research included medical specialists categorized as basic specialists, medical support, other medical specialists, and subspecialists according to the Ministry of Health Regulation No. 30/2019 [37]. The sample consisted of 341 medical specialists working and residing in remote areas, selected through purposive (non-probability) sampling. Direct questionnaires were distributed to medical specialists in 10 selected provinces.

### Data analysis

#### Descriptive statistics

The respondent profiles were analyzed using descriptive statistics to provide details about their characteristics. Descriptive statistical analysis was relevant to determine the distribution of respondents based on their demographic characteristics.

#### Conditional logit regression and group analysis

We utilized conditional logit regression to estimate preferences in the DCE retention questions. The analysis was conducted for the entire sample and also for groups based on placement and employment status. Positive coefficients indicated a preference for a particular attribute level compared to the reference level. Odds ratios (OR) were used to assess the likelihood of liking or disliking attribute levels compared to its reference level.

#### Willingness to Accept (WTA) analysis

The WTA analysis was conducted to estimate the compensation medical specialists desired for changes in non-monetary attributes. The Marginal Willingness to Accept (mWTA) values, expressed in Indonesian rupiah (IDR), represent the amount of compensation respondents are willing to accept due to changes in attribute levels. Higher mWTA values indicate a higher compensation that respondents desire for specific attribute levels. The mWTA calculation is based on the assumption of maximizing respondent utility and is expressed as follows [38]:

$$mWTA=\frac{Coefficient\;of\;non-monetary\;}{Coefficient\;of\;monetary}$$

### Ethical clearance

This study received ethical approval from the Faculty of Nursing, Universitas Airlangga ethical committee, ensuring the protection of human rights and welfare in health, under the reference number 2682-KEPK. Participants, all of whom were adults, were fully informed about the study’s objectives, procedures, potential risks, and benefits before their participation. Informed consent was obtained from participants through both verbal and written means. Verbal consent was documented to ensure transparency and understanding. Participants provided their informed consent by signing the informed consent form, thereby indicating their willingness to take part voluntarily.

## Results

A total of 341 respondents were spread across 10 remote areas in Indonesia. The profile of respondents is reported in Table 2. It can be seen that the highest number of respondents was from North Sumatra (17.30%). Most (53.96%) were men and 97.07% were graduates of public universities. Almost all (88.27%) were married with two or more children (61.00%). Additionally, 64.52% were not born in rural or remote areas. The majority were civil servants (73.61%) with over five years of work experience (66.57%). Respondents mostly came from one province with 195 placement locations (57.18%). The average age of the respondents was 29–40 years (49.85%). These demographic details laid the foundation for analysis of medical specialist retention factors.

**Table 2:** Profile of respondents.

Variable		n	%
Age	29–40 years	170	49.85
	41–52 years	161	47.21
	53–64 years	10	2.93
Gender	Female	157	46.04
	Male	184	53.96
Marital Status	Widowed/Divorced	9	2.64
	Single	31	9.09
	Married	301	88.27
Number of children	Have no children as yet	57	16.72
	Have 1 child	76	22.29
	Have 2 or more children	208	61.00
Place of birth	Not born in a rural/remote area	220	64.52
	Born in a rural/remote area	121	35.48
College graduates	Private college	10	2.93
	State college	331	97.07
Job status	Non-civil servant	90	26.39
	Civil servant	251	73.61
Work experience	<1 year	35	10.26
	1–2 years	40	11.73
	2–3 years	17	4.99
	3–4 years	22	6.45
	Above 5 years	227	66.57
Current placement	Same province as region of origin	195	57.18
	Different Province as region of origin	146	42.82
Province	Maluku	40	11.73
	North Maluku	24	7.04
	West Nusa Tenggara	32	9.38
	East Nusa Tenggara	49	14.37
	Papua	10	2.93
	West Papua	33	9.68
	Central Sulawesi	34	9.97
	West Sumatra	32	9.38
	North Sumatra	59	17.30
	Lampung	28	8.21

Table 3 presents the estimation results from the conditional logit of medical specialist retention. The results of the analysis showed that of the eight attributes that had been determined, there was one that was not significant, namely the number of cases handled each year. In terms of the location attribute, medical specialists exhibited a preference for non-remote areas over remote areas, with an odds ratio of 0.74. On the attribute of medical facilities, the highest preference regarding attributes of medical facilities was in locations with more than standard medical facilities with an odds ratio of 2.35. In the attribute of incentive sources, respondents had positive preferences for being maintained with all incentives with an odds ratio of 1.95 compared to other incentive. In terms of the continuing professional development program attribute, they exhibit a higher preference for fully funded programs with an odds ratio of 2.84. With regards to the security attribute, medical specialists highly valued security guarantees from the local government with an odds ratio of 6.11. In the length of commitment attribute, medical specialists were unwilling to work in the same place until retirement with an odds ratio of 0.66. The coefficient for net income indeed indicates a positive value, suggesting a preference for the highest monetary income.

**Table 3:** Estimation of conditional logit retention of specialists for all samples (n = 341).

Attribute	Level	Coef (SE)	Odds ratio	mWTA (IDR)
Location	Remote areas (not remote areas ^R^)	-0.2887*** (0.0853)	0.7492	10,784,460
Medical facility	Adequate to standard (inadequate ^R^)	0.5101*** (0.0786)	1.6650	19,054,912
	More than standard (inadequate ^R^)	0.8555*** (0.1160)	2.3530	31,957,415
Source of incentives	Medical services or services (Regional incentives/central incentives ^R^)	0.5840*** (0.0805)	1.7930	21,815,465
	All incentives (Regional incentives/central incentives ^R^)	0.6726*** (0.0705)	1.9590	25,125,140
Continuing professional development program	Partially funded (unfunded ^R^)	0.5273*** (0.0562)	1.6940	19,697,422
	Fully funded (unfunded ^R^)	1.0450*** (0.0950)	2.8450	39,036,235
Security	Security guarantees are available from local customs/communities (no guarantee of security from the government ^R^)	1.6590*** (0.0849)	5.2540	61,972,357
	There is a security guarantee from the local government (no security guarantee from the government is available yet ^R^)	1.8110*** (0.1132)	6.1140	67,650,355
Length of commitment	Willing to work in the same place for 4 years (Willing to work in the same place for 2 years ^R^)	0.4585*** (0.0692)	1.5820	17,127,381
	Willing to work in the same place until retirement (willing to work in the same place for 2 years ^R^)	-0.4072*** (0.1147)	0.6655	15,211,057
Number of cases handled	Adequate (little ^R^)	-0.0287 (0.1064)	0.9717	1,072,843
	many (little ^R^)	-0.1291 (0.0912)	0.8789	4,822,563
Net income		0.0000*** (0.0000)	1.0000	

Note: IDR: Indonesian Rupiah;SE: Standard Error

***, **, and * denote that the parameters are different from zero at the 1%, 5%, and 10% significance levels

^R^ = Reference level.

## Discussion

The study found that the preference of medical specialists to work in remote areas of Indonesia was influenced by various kinds of interventions, both financial and non-financial. Security emerged as the top attribute in this study, with specialists valuing guarantees from both the regional government (OR = 6.1; Coef = 1.8) and local security (OR = 5.3; Coef = 1.7). If assessed based on the mWTA value, medical specialists were willing to pay Rp. 67,650,355 to get a security guarantee from the local government, indicating that security guarantees were highly valued by specialists, so they were willing to spend that amount of money to guarantee their safety. Several studies have also shown that security attributes support the work safety of medical specialists at the individual level [22, 23, 39]. The government has taken steps to address security concerns, introducing the Mandatory Service for medical specialists (Wajib Kerja Dokter Spesialis or WKDS) program in 2017 through Presidential Regulation Number 4 of 2017 [40]. This program not only ensures the welfare of medical specialists but also underscores the importance of their safety and security [41]. However, a healthcare service report in Papua and West Papua provinces (2022) identified security challenges as a persistent issue impacting the willingness of medical specialists to work in remote areas [42, 43]. Despite central government security guarantees, regional regulations and collaboration with local security are crucial in addressing security problems faced by medical specialists in remote areas. Effective security guarantees for medical specialists necessitate collaborative efforts between local governments and security entities, aligning with the broader goal of ensuring the safety of healthcare professionals in remote areas.

Continuing professional development programs were an attribute that was also considered crucial to retaining medical specialists in remote areas. The results of this assessment indicated that medical specialists wanted continuing professional development programs to be fully funded by the Indonesian government (OR = 2.8; Coef = 1.05), compared to having to pay for these themselves. If assessed based on a negative mWTA value, specialists were willing to pay IDR. 39,036,235 to participate in a fully funded program. These results suggest that fully funded programs were highly valued by medical specialists, such that they are willing to spend that amount of money to participate in the program. Continuing professional development programs are seen as important for maintaining competency and improving performance among all health workers in order to provide a quality level of service [23, 44, 45]. Continuing professional development programs can also support medical specialists in remote areas to communicate with each other in maintaining social and professional networks [23, 45]. Medical specialists often have difficulty accessing such programs because they are far from the program location and program organizers. Implementation with digital platforms is also often constrained by technicalities such as unreliable connectivity [46]. The Indonesian Ministry of Health has conducted continuing professional development programs regularly and implemented these in technical units in each province. Activities are also carried out offline, online, or hybrid with several financing categories namely independent, fully funded, and partially funded [47]. The combination of continuing professional development programs and other interventions is expected to increase the retention of medical specialists in remote areas.

Collaborative efforts present a promising solution to these challenges. Previous collaborations, such as the Academic Health System (AHS) jointly implemented by the Ministry of Education, Culture, Research, and Technology in conjunction with the Ministry of Health, aim to expedite capacity and quality improvement in medical faculties. The goal is to produce physicians and medical specialists who can enhance healthcare services [48]. However, specific collaborations focused on enhancing the capacity of medical specialists through Continuous Professional Development (CPD) programs have not been established to date. To address this gap, a potential collaboration with the Ministry of Communication and Information could introduce online CPD programs, ensuring stable and affordable internet access, even in remote areas. Professional organizations, such as the Indonesian Doctors Association (IDI) and other specialist professional organizations can also contribute to successful programs. For example, incorporating these organizations into CPD projects could bring extra resources, expertise, and networks necessary for the implementation. This initiative could be a part of a broader effort to enhance the implementation of CPD programs, reaching all medical specialists, particularly those in underserved regions.

Medical facilities (OR = 2.4; Coef = 0.9) are also an attribute that was sufficiently considered in the retention of medical specialists. The results of this assessment indicate that medical specialists preferred to work in areas with facilities exceeding standard adequacy. If rated based on a negative mWTA value, medical specialists were willing to pay Rp. 31,957,415 to be placed in higher-quality health facilities. This demonstrates that medical specialists valued excellent health facilities and were willing to spend that amount of money to work in facilities with higher standards. Medical facilities are closely related to the quality of care and readiness to practice. Medical specialists without adequate medical facilities will experience limitations in providing health services to patients [24, 49, 50]. In addition, medical facilities can affect people’s confidence in their ability to produce a specified level of performance in disadvantaged areas [31, 49, 50]. The lack of medical facilities in hospitals has an impact on the quality of services provided by medical specialists to patients [51]. Effective problem-solving requires cross-sectoral collaboration, particularly between the Ministry of Health, the Ministry of Home Affairs, and also the regional/subnational government. Simply providing standard facilities and equipment may not sufficiently address the complex challenges faced by medical specialists. Our study emphasizes the need for collaborative initiatives involving multiple stakeholders to achieve comprehensive solutions. It underscores that a holistic approach, involving various government agencies, is essential for retaining medical specialists in Indonesia’s remote locations.

The "hospital-based residency program" recently announced by the Ministry of Health intends to solve Indonesia’s medical specialized shortages and disparity. This program provides specialist medical education program under the hospital oversees with financial support from the central government, and uses a curriculum established with national and international competence to meet high standards. It prioritizes medical specialists from remote areas, with placements based on prioritizing areas after graduation [52]. While this project is critical for increasing the availability and distribution of qualified medical specialists throughout Indonesia, further evaluation is required to assess and ensure their retention in remote areas.

This study provides important insights regarding the retention of medical specialists in remote areas. The strength of this study is its emphasis on aligning retention efforts with existing regulations and analyzing the numerous elements that influence these restrictions. The present approach provides a more comprehensive understanding of the problem than studies that have focused solely on a single aspect. However, it is also crucial to understand the research’s limitations, particularly when non-probability sampling approaches are used. Although this method may make data collecting more effective, it may limit the generalizability of findings to the broader population of medical specialists in remote locations.

## Conclusion

Our DCE study highlights the imperative need for a comprehensive intervention package, primarily emphasizing non-financial factors. For instance, medical specialists exhibited preference for non-remote areas, locations with more than standard medical facilities, and fully funded professional development programs. Security guarantees from the local government also emerged as a crucial factor. This strategic combination can significantly contribute to medical specialist retention. The interconnected nature of these interventions, adapted to the local context, forms a holistic approach to address the unique challenges faced by medical specialists in remote areas. We argue that existing regulations should extend beyond utilization considerations to encompass retention aspects within a cohesive policy framework. This study strongly advocates for policies that not only attract but, more importantly, retain medical specialists in remote regions. Looking ahead, future research should explore family-related variables, investigate the educational backgrounds of family members, and explore psychological aspects influencing medical specialists to further refine intervention strategies.
